# Computational and Functional Analysis of Structural Features in the ZAKα Kinase

**DOI:** 10.3390/cells12060969

**Published:** 2023-03-22

**Authors:** Valdemar Brimnes Ingemann Johansen, Goda Snieckute, Anna Constance Vind, Melanie Blasius, Simon Bekker-Jensen

**Affiliations:** Center for Healthy Aging, Department of Cellular and Molecular Medicine, Faculty of Health and Medical Sciences, University of Copenhagen, Blegdamsvej 3B, DK-2200 Copenhagen, Denmark

**Keywords:** ribotoxic stress response, ZAKα, JNK, p38, ribosomes, translation, YEATS domain

## Abstract

The kinase ZAKα acts as the proximal sensor of translational impairment and ribotoxic stress, which results in the activation of the MAP kinases p38 and JNK. Despite recent insights into the functions and binding partners of individual protein domains in ZAKα, the mechanisms by which ZAKα binds ribosomes and becomes activated have remained elusive. Here, we highlight a short, thrice-repeated, and positively charged peptide motif as critical for the ribotoxic stress-sensing function of the Sensor (S) domain of ZAKα. We use this insight to demonstrate that the mutation of the SAM domain uncouples ZAKα activity from ribosome binding. Finally, we use 3D structural comparison to identify and functionally characterize an additional folded domain in ZAKα with structural homology to YEATS domains. These insights allow us to formulate a model for ribosome-templated ZAKα activation based on the re-organization of interactions between modular protein domains. In sum, our work both advances our understanding of the protein domains and 3D architecture of the ZAKα kinase and furthers our understanding of how the ribotoxic stress response is activated.

## 1. Introduction

The MAP kinases (MAPK) p38 and JNK are central transducers of cellular stress pathways [1]. In mammals, the p38 family comprises four members (p38α, p38β, p38γ, and p38δ), whereas the JNK family comprises three members (JNK1, JNK2, and JNK3). They are activated by a number of stress agents such as UV light, oxidative stress, and heat or osmotic shock [1]. In addition, MAPKs are activated by a number of extracellular signaling molecules such as growth factors, hormones, and cytokines [2]. MAPKs are generally activated through signal transduction cascades involving upstream MAP kinase kinases (MAPKKs) and MAP kinase kinase kinases (MAPKKKs). In stress responses, p38 largely works as a “master kinase” that phosphorylates and activates at least ten different downstream kinases, including members of the MAPKAP kinase family, Msk, and Mnk kinases [3,4,5]. These kinases in turn phosphorylate a plethora of substrates that impact the functionality of diverse cellular processes such as cell cycle progression [6], cell differentiation [7], transcription [8], and protein trafficking [9], to mention a few. JNK kinases, on the other hand, do not appear to amplify their signaling through downstream kinases. JNK kinases are linked to apoptotic signaling [10], cell differentiation [11], and metabolic regulation [12]. Taken together, the elaborate signaling events initiated by p38 and JNK help cells to adequately respond to changing environmental conditions.

For extracellular ligand-mediated MAPK activation, the signaling from upstream MAPKKKs has been largely deduced. However, how the same group of kinases respond to stressful conditions is less understood. An example of this is the Ribotoxic Stress Response (RSR), where the MAPKKK ZAKα is activated by translational impairment and/or dysfunctional ribosomes [13]. Multiple triggers of RSR signaling have been identified. These include small-molecule ribosome inhibitors, e.g., anisomycin, that inhibits translation by binding the peptidyl transferase center of the ribosomal 60S subunit [14]. Another example is that of the ribotoxic enzymes of microbial origin, e.g., ricin, Shiga toxin, and α-sarcin, which either depurinate or cleave the sarcin-ricin loop (SRL) of the 28S rRNA [15]. Furthermore, some chemotherapeutics (e.g., doxorubicin) and UV-irradiation are recognized as ribotoxic agents [13,16], the latter due to crosslinking of nucleotides in mRNA templates [17]. Recently, translational down-regulation following nutrient depletion and amino acid starvation were also demonstrated to be potent ZAKα-activating stimuli [18]. ZAKα homologues are absent from unicellular organisms, such as the yeast *Saccharomyces cerevisiae*, but clearly present in the genomes of vertebrate species, including fish and mammals. Curiously, only some metazoans, such as *Caenorhabditis elegans*, have a ZAKα homologue, whereas others such as *Drosophila melanogaster* do not.

ZAKα is a highly modular protein consisting of a number of annotated domains. Of these, crystal structures have determined the folding of the N-terminal kinase, leucine zipper, and SAM domains. Point mutations in the Sterile Alpha Motif (SAM) of ZAKα have been identified as a cause of developmental limb defects in humans and mice [19]. Surprisingly, patient-derived mutations that disrupt the folding of the SAM domain are associated with constitutive activation, and not loss-of-function, of ZAKα [20]. These insights suggest that the SAM domain is a gatekeeper for harnessing ZAKα activity. In addition to the above, ZAKα harbors two partially redundant ribosome-binding domains required for its activation [20]. These have been dubbed the Sensor (S) and C-terminal (CTD) domains and are located within a C-terminal ribosome-binding region (RBR). The roughly 25 amino acid-long CTD constitutes a positively charged region that interacts with an RNA component of the ribosome, presumably a helix of 18S rRNA that is exposed in the intersubunit space. The deletion or charge-neutralizing mutation of this region only has a marginal effect on the ZAKα activation potential. However, when combined with the deletion of the S domain, ZAKα is completely refractory to activation upon a range of ribotoxic stress-inducing stimuli. The S domain, on the other hand, is much less characterized, with the domain boundaries only being loosely defined and the identity and nature of its binding partner remaining obscure. As is the case with the CTD, isolated deletion of the S domain does not impair ZAKα activation [20].

Some knowledge about the mechanism(s) underlying ZAKα activation has been gleaned in recent years, such as the ability of the kinase to bind directly to ribosomes through dedicated sensor domains [20] and become activated by collided [17] as well as stalled ribosomes [18]. However, the ability of the kinase to discriminate between ribosomal states remains unexplained, in part because stable ribosome-ZAKα complexes have escaped structural elucidation by cryo-EM. Here, we reveal a short and thrice-repeated peptide motif as the key structural feature that governs the functionality of the S domain. Using computational approaches, we also describe an additional folded domain (“YEATS-Like Domain/YLD”) immediately downstream from the SAM domain that is required for the full activation of ZAKα. Our work offers important new insights into the structural features that determine the ribosome-templated activation of this important kinase.

## 2. Materials and Methods

### 2.1. Cell Culture and Reagents

Human osteosarcoma cells (U2OS, ATCC, HTB-96) were cultured in Dulbecco’s Modified Eagle’s Medium (DMEM, Biowest, Nuaillé, France) supplemented with 10% fetal bovine serum (FBS, Sigma Aldrich, St. Louis, MO, USA), L-glutamine, 1% penicillin, and streptomycin and cultured at 37 °C in a humidified 5–8% CO_2_ cell incubator. Experiments were conducted when the cells reached 70–80% confluency. U2OS ΔZAK cells were generated and described previously [20]. To generate cell lines stably expressing WT, truncations, and other mutants of ZAKα under the control of doxycycline-inducible promoters, cells were transfected with pcDNA4/TO/Strep-HA-ZAKα constructs and pcDNA6/TR (Life Technologies, Carlsbad, CA, USA) in a 1:4 ratio and selected for 14 days with zeocin (200 μg/mL) and blasticidin (5 μg/mL) (both Thermo Fisher Scientific, Waltham, MA, USA). Individual clones were picked, and expression was analyzed by immunofluorescence and Western blotting. UV-B light (500 J/m^2^) was delivered to cells in a BS-02 irradiation chamber equipped with 254 nm bulbs (Gröbel Elektronik, Ettlingen, Germany). Cells were subsequently allowed to recover for 1 h prior to harvesting. The chemicals used in this paper were: doxycycline (Sigma-Aldrich, D3347, 0.13 μg/mL, overnight), anisomycin (Sigma-Aldrich, A9789, 1 μg/mL, 1 h), and Earle’s Balanced Salt Solution (EBSS) (Sigma-Aldrich, #E3024, 18 h). 

### 2.2. Western Blotting and Antibodies

For protein extraction, cells were lysed in EBC lysis buffer (pH 7.5, 1 mM EDTA, 0.5% NP-40, 50 mM Tris buffer, 150 mM NaCl) supplemented with protease inhibitors (protease inhibitor cocktail (Roche, Basel, Switzerland)), phosphatase inhibitors (sodium orthovanadate, β-glycerolphosphate, and sodium fluoride), desumoylase and deubiquitinase inhibitors (N-ethylmaleimide), as well as a reducing agent (dithiothreitol). Laemmli sample buffer was added to the whole cell extracts prior to 10 min of boiling at 95 °C. Samples were subsequently resolved by SDS-PAGE and transferred to nitrocellulose membranes. Membranes were then blocked in PBS-T + 5% milk prior to incubation with primary antibody overnight at 4 °C. Membranes were washed in PBS-T 5 times for 5 min and incubated with HRP Goat Anti-Rabbit or Goat Anti-Mouse IgG Antibody (H + L) for 1 h at room temperature. Membranes were washed 3 times for 5 min in PBS-T and visualized by chemiluminescence (Clarity Western ECL substrate, Bio-Rad, Hercules, CA, USA) using the Bio-Rad ChemiDoc imaging system. To immunoblot for various proteins on the same membrane, reblotting was performed by stripping nitrocellulose membranes with 1× Re-Blot Plus Strong Solution (Millipore, Burlington, MA, USA) for 15 min at room temperature. The membranes were subsequently washed 3 times for 5 min in PBS-T, blocked as previously described, and incubated with a new primary antibody at 4 °C overnight. Antibodies used in this paper were: anti-phospho-p38 (Cell Signaling, #9216 and #4511S, Danvers, MA, USA), anti-p38 (Cell Signaling, #9212), anti-phospho-SAPK/JNK (Cell Signaling Technology, #9255), anti-ZAKα (Bethyl #A301-993A, Montgomery, TX, USA), anti-p150 (BD biosciences, #610473, Franklin Lakes, NJ, USA), ⍺-tubulin (Sigma, #T9026), anti-phospho-p70S6K (Cell Signaling, #9234), and anti-hemagglutinin (Santa Cruz, #Sc-7392, Dallas, TX, USA).

### 2.3. Cloning and Plasmids

Constructs carrying deletions, internal mutations, or both were ordered as synthetic genes (GeneArt). Constructs were then PCR-cloned into pcDNA4/TO/Strep-HA. U2OS ΔZAK stably expressing ZAKα WT and ZAKα ΔCTD were generated previously [20]. Plasmid transfections were performed using polyethylenimine (PEI, 3.1 ng/μL–4.4 ng/μL, 4 h) or FUGENE6 (Promega, Madison, WI, USA) according to manufacturer’s protocol when cells were 40–50% confluent. 

### 2.4. Bioinformatics

PDB text file formats of ZAKα domains were prepared in PyMOL from AF-Q9NYL2-F1 prior to computational distanced-assisted matrix alignment (DALI) analysis. DALI is a server for 3D protein structure comparison [21,22], and recent upgrades of the server include the foldomes of key organisms in the AlphaFold Database (version 1) [23]. The text file format was prepared to prevent topological output hits based on other domains than the domain of interest to obtain the most reliable Z-score [24] and because DALI only accepts PDB text file formats as input. In DALI, we performed a pairwise hierarchical AlphaFold database search that compared the PyMOL-prepared ZAKα YLD against the human subset of the database. All parameters for this search were set to default as previously described [23,24]. Primary and secondary alignments, overlays, and Z, RMSD, and LALI scores were generated in the DALI webserver. Overlays were visualized and inspected with PyMOL. Protein domain indications of DALI hits were schematized based on Pfam annotations in DALI taken from the current reference database for protein domains Pfam 35.0 [23] that contains 19,632 protein families for structures in PDB and AlphaFold. YEATS domain Pfam source is PF03366. SWIB/MDM2 domain Pfam source is PF02201. PAE plot of the predicted structure of ZAKα was generated with AlphaFold. The code of the python script for PAE plotting directly from AlphaFold is available at Github (see Data Availability Statement). 

## 3. Results

### 3.1. Short Peptide Motifs Underlie Functionality of the Sensor Domain in ZAKα

**Figure 1:**
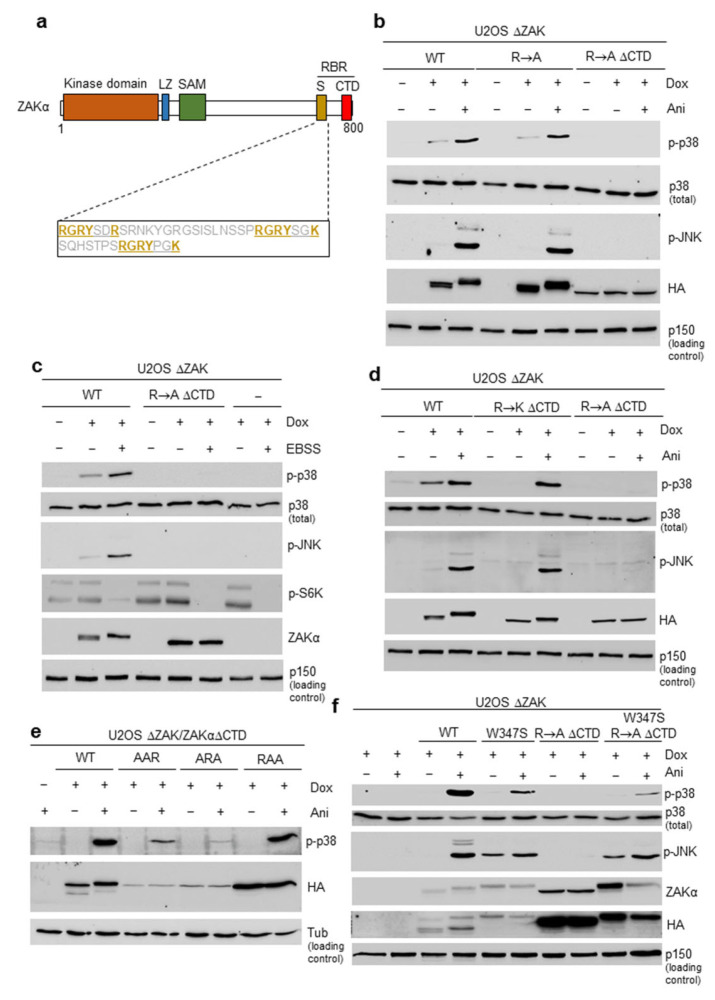
**Redundant peptide arrays underlie functionality of the sensor domain in ZAKα**. (**a**) Schematic of the ZAKα protein highlighting the three RGRYXXR/K motifs in the sensor (S) domain. ZAKα S domain mutants used in this study are detailed in Figure S1a. LZ, Leucine Zipper; SAM, Sterile Alpha Motif; S, Sensor Domain; CTD, C-Terminal Domain; and RBR, Ribosome Binding Region. (**b**) U2OS cells deleted for ZAK (U2OS ΔZAK) were rescued with either STREP-HA-tagged wildtype (WT) ZAKα, a version of ZAKα with all arginines and lysines mutated to alanine in RGRYXXR/K motifs (R → A), or a version of R → A combined with a deletion of the CTD (R → A ΔCTD). Expression was induced by doxycycline (Dox) and cells were treated with anisomycin (Ani—10 μg/mL, 1 h). Lysates were analyzed by immunoblotting with the indicated antibodies. (**c**) U2OS ΔZAK cells were rescued with WT or R → A ΔCTD as in (**b**) and were incubated in EBSS medium (18 h). Lysates were analyzed as in (**b**). (**d**) U2OS ΔZAK cells were rescued with either WT, R → A ΔCTD, or a version of ZAKα with the corresponding lysine substitution mutant (R → K ΔCTD). Cells were induced for ZAKα expression, treated with anisomycin, and analyzed as in (**b**). (**e**) U2OS ΔZAK cells were rescued with mutants of ZAKα from (**a**), containing only one functional RGRYXXR/K peptide. Cells were treated and analyzed as in (**b**). (**f**) U2OS ΔZAK cells were rescued with ZAKα constructs combining R → A ΔCTD mutation with a disease-causing point mutation in the SAM domain (W347S) that is associated with constitutive ZAKα activity or a version of W347S combined with R → A ΔCTD (W347S R → A ΔCTD). Cells were treated and analyzed as in (**b**). p-, phosphorylated (activated) form of kinase; Tub, Tubulin.

In previous work, the functionality of the S domain of ZAKα was demonstrated by internal deletion of a region spanning 150 amino acids [20]. Upon analysis of the sequence of this domain, we noticed the presence of three closely spaced peptide motifs with the consensus sequence RGRYXXR/K (Figure 1a).

We set out to investigate if these motifs are of functional importance for the ability of the S domain to sense ribotoxic stress insults. To this end, we rescued previously established *Zak* KO U2OS (U2OS ΔZAK) cells with doxycycline-inducible versions of ectopic STREP-HA-tagged ZAKα, where all arginines and lysines in these motifs were replaced with alanines, either in a full-length context or with a deletion of the last 27 amino acids of the CTD (R -> A, R -> A ΔCTD) (Figure S1a). Mutation of the S domain in isolation (R -> A) did not abrogate anisomycin-induced activation of ZAKα, as visualized by the phosphorylation of the two downstream kinases p38 and JNK (Figure 1b). However, when combined with CTD deletion, which on its own does not preclude ZAKα activation [20], this response was completely absent (Figure 1b). This double mutant was also defective for the characteristic anisomycin-induced gel mobility shift (Figure 1b), which is indicative of ZAKα autophosphorylation [20]. We recently showed that cells incubated in the starvation medium Earle’s Balanced Salt Solution (EBSS) also activate p38 and JNK in a ZAKα-dependent fashion [18]. Similar to the anisomycin treatment above, this response was also dependent on the integrity of the S domain peptide motifs within ZAKα (Figure 1c). As a control for starvation-associated mTOR inhibition, we probed the phosphorylation state of S6 kinase (p-S6K) [25]. The arginines within the RGRYXXR/K motifs are subject to methylation [26] (phosphosite.org (accessed on 16 March 2023)). In order to investigate whether the positive charge of these residues or their modification is more relevant, we also constructed a CTD-deleted version of ZAKα where we changed all the relevant arginines to lysines (R -> K ΔCTD) (Figure S1a). This mutant perfectly rescued U2OS ΔZAK cells (Figure 1d), suggesting that arginine methylation within these motifs is not required for ZAKα activation. We also generated an alanine substitution construct where only a single of the three motifs retained their arginine residues (AAR, ARA and RAA, Figure S1a). Two of these (AAR and ARA) displayed low protein stability and/or were inefficiently expressed, resulting in the poor rescue of U2OS ΔZAK cells (Figure 1e). However, the RAA ΔCTD mutant perfectly rescued ZAK deficiency (Figure 1e), indicating that only a single intact RGRYXXR/K motif is sufficient to confer full functionality on the S domain. Our results highlight a repetitive peptide motif in ZAKα that is critical for the ribotoxic stress-sensing function of the S domain.

### 3.2. Mutation of the SAM Domain Bypasses the Requirement of Ribosome Binding Domains for ZAKα Activation

We next combined inactivating mutations in the S and CTD domains (R -> A ΔCTD) with a previously described point mutation (W347S) in the SAM domain that confers constitutive activity and mild instability on ZAKα [20]. Strikingly, W347S mutation bypassed the requirement of the two ribosome-binding domains for ZAKα activity and downstream activation of p38 and JNK. First, doxycycline-induced expression showed that both the W347S mutant and the R -> A ΔCTD W347S composite mutant were already shifted in SDS-PAGE prior to anisomycin treatment, indicating autophosphorylation of the kinase (Figure 1f—compare lanes 3, 4, 5, and 9 of the ZAKα blot). Second, doxycycline induction of these two mutants resulted in substantial background levels of JNK (and to a lesser extent p38) activity (Figure 1f—compare lanes 3, 5 and 9, of the p-JNK blot). These results indicate that the SAM domain is critical for maintaining ZAKα in an inactive, but activation-competent, state. The abolition of this negative mode of regulation results in a version of ZAKα that no longer strictly requires communication with the ribosomes via the S and CTD domains for activation. Anisomycin-treatment of W347S- and R -> A ΔCTD W347S-expressing cells still increased the phosphorylation of JNK and p38 (Figure 1f), indicating the potential presence of additional or alternative ribosome-sensing regions in ZAKα. 

### 3.3. ZAKα Contains a YEATS-Like Domain with High Topological Similarity to Annotated YEATS Domains

**Figure 2:**
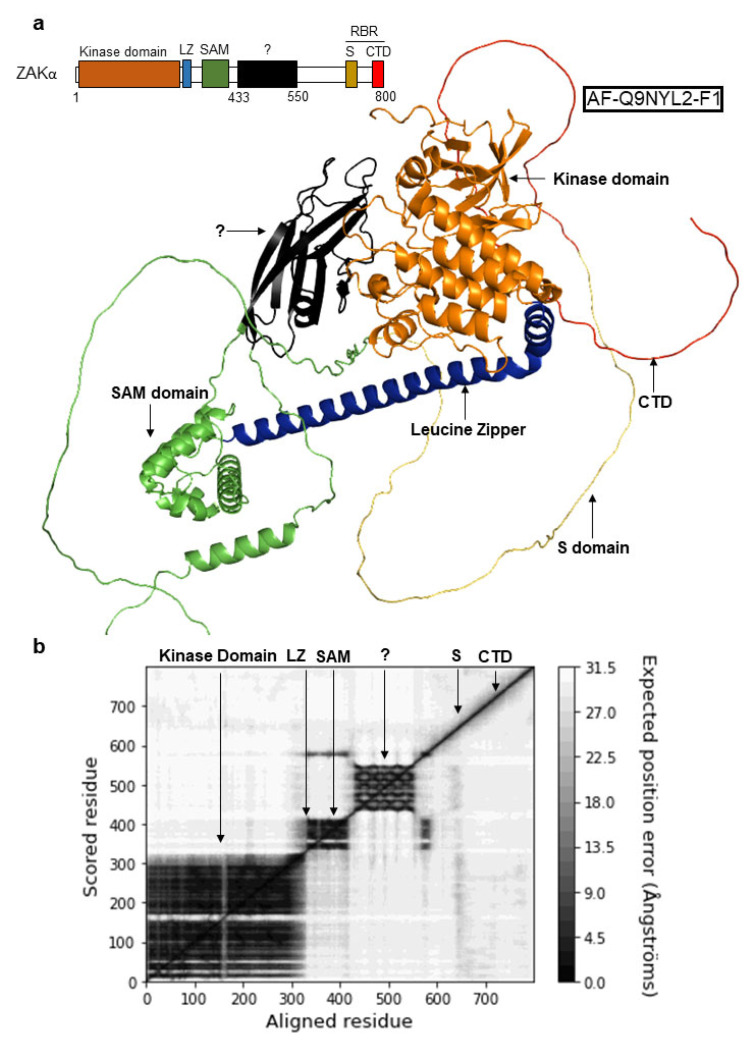
**AlphaFold predicts the presence of an unannotated structural domain in ZAKα.** (**a**) AlphaFold-predicted three-dimensional structure of human ZAKα (AF-Q9NYL2-F1 model version 1 of PDB entry Q9NYL2). Annotations of annotated and unannotated structurally predicted domains correspond to the schematic (top left). (**b**) Predicted aligned error (PAE) plot of residue pairs from the AlphaFold database. The plot was made from the AlphaFold JSON PAE file by creating an 800 × 800 array with the PAE values for each amino acid residue pair. Folded protein domains are indicated by black arrows. LZ, Leucine Zipper; SAM, Sterile Alpha Motif; S, Sensor Domain; CTD, C-Terminal Domain; and RBR, Ribosome Binding Region.

Despite recent advances in the search for ZAKα activation mechanism(s) [17,20], much remains to be elucidated about the molecular architecture and functional domains of the kinase. This can partially be explained by the fact that only positions 5-309 of ZAKα, encompassing the kinase domain and part of the leucine zipper, have been structurally solved by X-ray crystallography [27,28,29]. We are thus lacking knowledge about structural features and/or domains in the C-terminal part of the protein, downstream of the SAM domain. We examined the AlphaFold-predicted folding of full-length ZAKα and noticed a putative folded domain, ranging from amino acid 433 to 550 (Figure 2a). The small linker region between the SAM domain and this hitherto unrecognized protein domain, as well as the sequences downstream, are predicted to be largely unfolded by AlphaFold. To assess confidence in the packing of the putative domain, as well as the large-scale topology of ZAKα, we examined the Predicted Aligned Error (PAE) of the model. This approach allowed us to visualize relative domain positions and explore the confidence of the AlphaFold prediction [30,31]. The putative domain, spanning amino acids 433 to 550 of ZAKα, displayed low PAE scores, suggesting that it is likely to fold as an independent domain (Figure 2b). 

**Figure 3:**
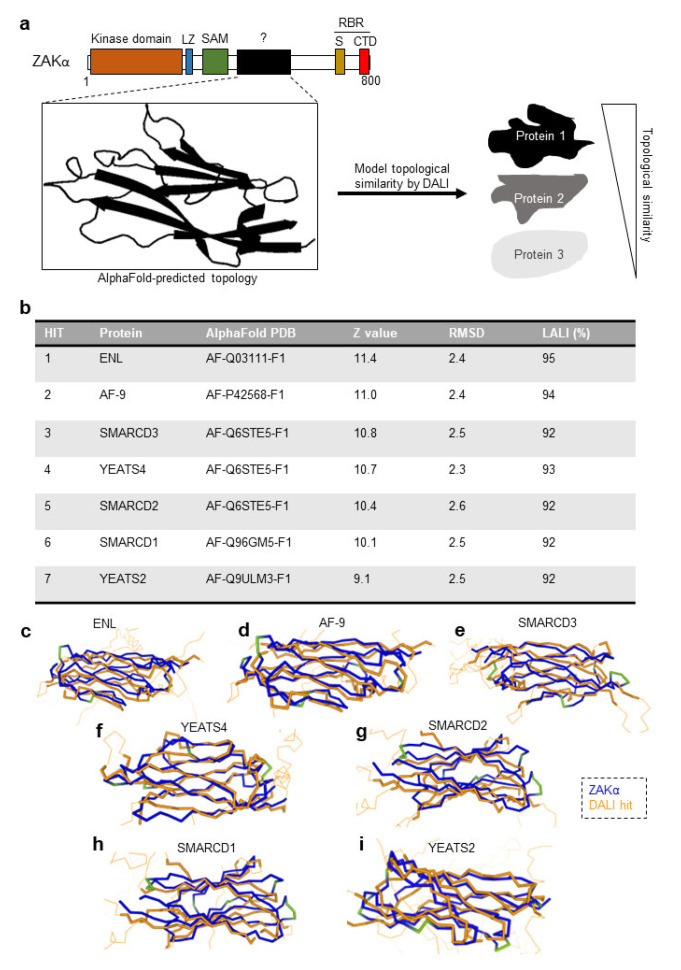
**The unannotated ZAKα domain shares topological similarities with other proteins.** (**a**) Schematic of the distance-assisted matrix alignment (DALI) method for structural comparison. A PDB file containing 3D coordinates of amino acids 433–550 in ZAKα was analyzed by the DALI web server for topological conservation against the human AlphaFold database. LZ, Leucine Zipper; SAM, Sterile Alpha Motif; S, Sensor Domain; CTD, C-Terminal Domain; and RBR, Ribosome Binding Region. (**b**) The top seven ZAKα-DALI hits (Z value > 9) from the hierarchical pairwise DALI analysis against the AlphaFold database. Z-values, root mean square deviation (RMSD), and number of equivalent residues (LALI) are listed. (**c**–**i**) Cα-Cα atom traces for amino acids 433–550 in ZAKα (blue/green) superimposed on DALI hits: ENL (**c**), AF-9 (**d**), SMARCD3 (**e**), YEATS4 (**f**), SMARCD2 (**g**), SMARCD1, (**h**) and YEATS2 (**i**) (all colored orange). Thick orange Cα-traces of the DALI hits represent traces that superimpose with the unannotated ZAKa domain, whereas thinner orange Cα-traces of the DALI hits represent unaligned parts of these proteins. The overlays were made in the DALI web server and inspected using PyMOL as described in Section 2.4. ZAKα traces are colored in a monochrome conservation color with blue indicating strong superimposition and green regions indicating poor superimposition on DALI hit structure.

We next sought to investigate whether this domain bears resemblance to known functional protein domains. A 2D-homology search did not return any hits, and we instead turned to a strategy of 3D-homology searches, looking for proteins with topological similarity. To this end, we subjected the uncharacterized ZAKα domain to pairwise, hierarchical distance-assisted matrix alignment (DALI) against the human AlphaFold database (Figure 3a). The DALI method measures the geometrical similarities between two structures, defined as the weighted sum of similarity in intramolecular distances of Cα-Cα traces [24]. The Z-score from DALI is based on an additive similarity function that integrates the maximization of equivalenced residues with the minimization of structural deviations. The Z-score accounts for the mean score, standard deviation, and average length of the two proteins (Figure S1b). Z-values of 8 and above are considered probable 3D-homologous relationships [24], where DALI hits indicate structural similarities that are unlikely to have arisen by chance. 

DALI analysis of the structure of the amino acids 433 to 550 of ZAKα against all human AlphaFold-predicted structure returned seven such hits (Figure 3b; Table S1). Four of these, ELN, AF-9, YEATS4, and YEATS2 harbor the so-called YEATS domain, and in all four cases, this domain was the region of 3D-similarity with residues 433 to 550 of ZAKα. The YEATS domain has an immunoglobulin-like fold [32] and is found in proteins involved in transcription and chromatin modification [33]. In several cases, YEATS domains have been characterized as readers of lysine modifications, most notably acetylation and crotonylation [34,35,36]. The other three hits were the three members of the SMARCD family, which are components of SWI/SNF-related ATPase and chromatin remodeling complexes [37]. The proportion of structurally equivalent residues between the ZAKα domain and the individual DALI hits were in the range of 90–95% (LALI score—Figure 3b). The superimposition of peptide backbones from pairwise alignments illustrated the high topological similarities of Cα traces between the putative ZAKα domain and the individual DALI hits (Figure 3c–i). The observed deviations in Cα traces (Figure 3c–i) account for the differences in root-mean-square deviation (RMSD) values (Figure 3b). 

**Figure 4:**
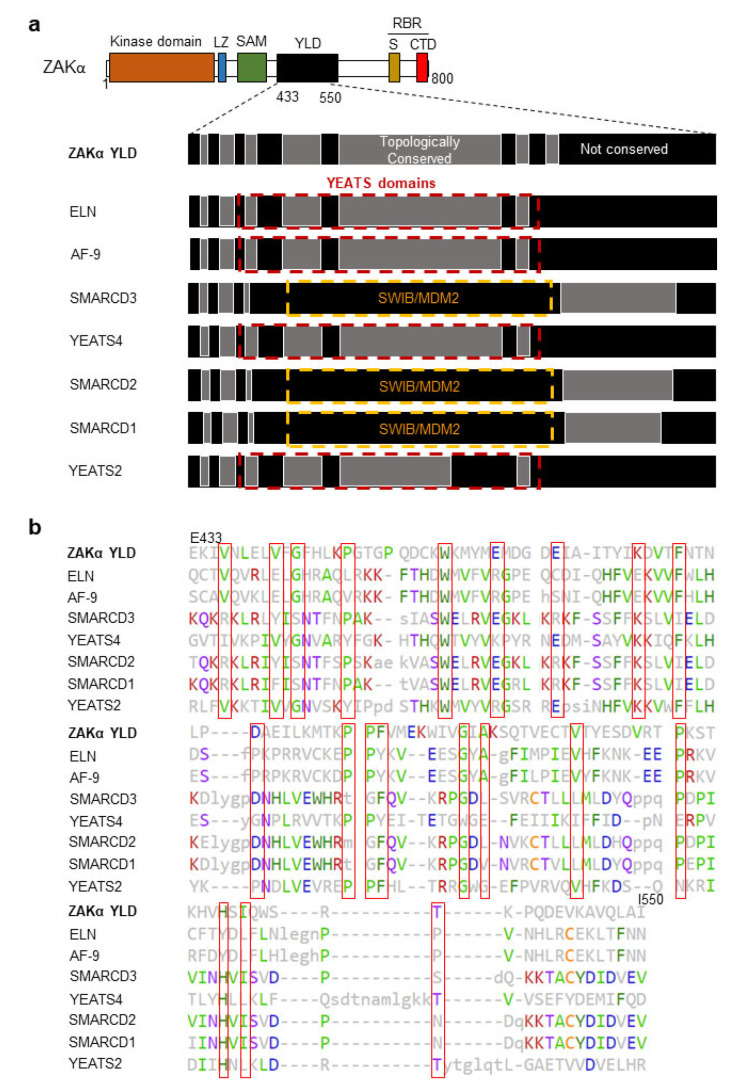
**ZAKα contains a YEATS-Like Domain with high topological similarity to annotated YEATS domains.** (**a**) The YEATS-Like Domain (YLD) of ZAKα shows high structural equivalence with YEATS-domains of hits from DALI analysis. Regions of high topological similarity (grey) are based on local Z-value scores when ZAKα YLD is superimposed on individual DALI hits. The methodology of Z-value score calculations is shown in Figure S1b. LZ, Leucine Zipper; SAM, Sterile Alpha Motif; YLD, YEATS-Like Domain; S, Sensor Domain; CTD, C-Terminal Domain; and RBR, Ribosome Binding Region. (**b**) Primary sequence alignment of ZAKα YLD and DALI hits from Figure 3. Gaps indicate unaligned regions. Uppercase letters denote structurally equivalent amino acids when compared to ZAKα YLD, whereas lowercase letters denote insertions relative to ZAKα YLD. The most frequent amino acid is colored in each column. Positions with a high conservation of specific amino acid residues are bracketed in red. Secondary sequence alignment is shown in Figure S1c.

We next investigated the regional, topological similarities between amino acids 433–550 of ZAKα and the DALI hits in Figure 3c–i. Strikingly, this putative domain within ZAKα shared high topology with the YEATS domains of ELN, AF-9, YEATS4, and YEATS2, suggesting a YEATS-Like Domain (YLD) within ZAKα (Figure 4a). 

The topological similarity of ZAKα YLD to the SMARCD proteins, however, did not map to the annotated SWIB/MDM2 domains of these proteins (Figure 4a). Multiple alignment of the ZAKα YLD domain with the DALI hits revealed only little primary sequence homology. However, multiple amino acid residues at specific positions were identical between the sequences (Figure 4b). Among these, glycines and prolines can enforce dihedral angles in the peptide backbone, which can be found in tight turns or β-bulge loops and are consistent with the predicted β-strand topology of the YLD and YEATS domains (Figure 2a and Figure 3a). Finally, we performed predictions of the contributions from individual amino acid residues to secondary structural features. This analysis uncovered a highly similar secondary structure contribution between the ZAKα YLD and the DALI hits (Figure S1c). Our bioinformatic approach that consisted of pairwise hierarchical DALI analysis against predicted structures in the human AlphaFold database thus highlights a putative folded domain in ZAKα with high similarity to YEATS domains.

### 3.4. Disruption of the YLD Decreases the Activation Potential of ZAKα

**Figure 5:**
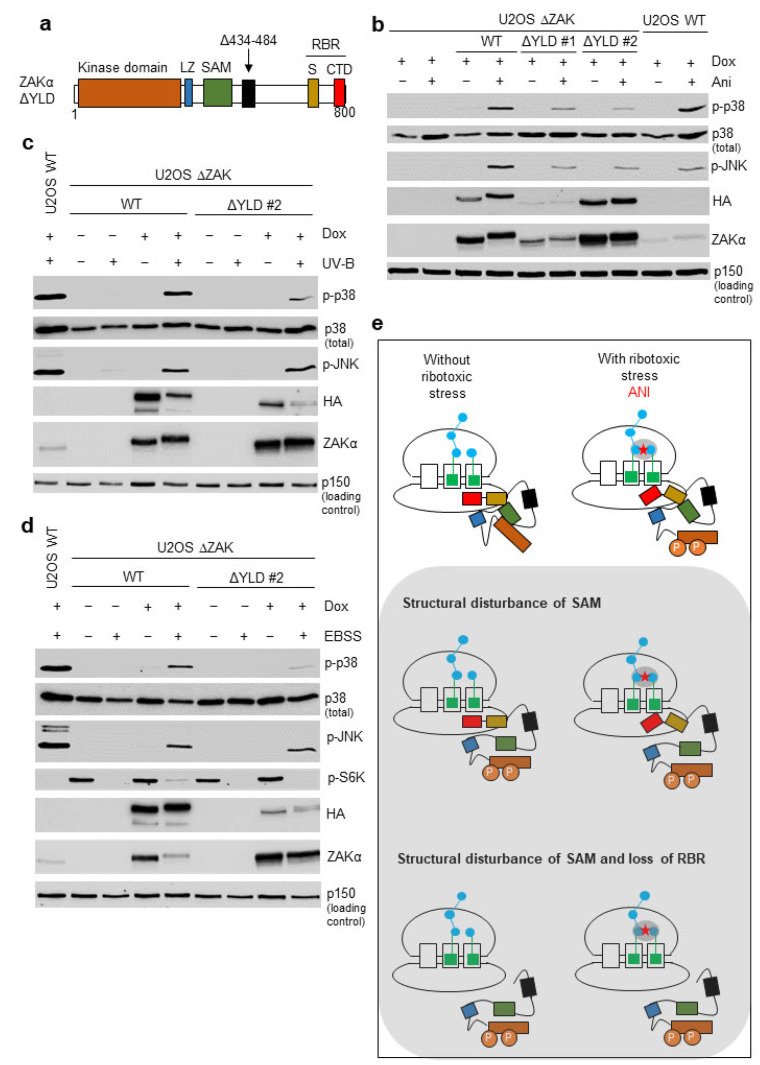
**Disruption of the YLD decreases the activation potential of ZAKα.** (**a**) Schematic of the ZAKα ΔYLD mutant protein, indicating a deleted region within the YLD domain. (**b**) U2OS ΔZAK cells were rescued with either wildtype (WT) ZAKα or ZAKα Δ YLD, of which two clones with different expression levels were analyzed (ΔYLD #1 and #2). Cells were induced for expression with doxycycline (Dox) and treated with anisomycin (Ani—10 μg/mL, 1 h). Lysates were analyzed by immunoblotting with the indicated antibodies. (**c**) Cells from (**b**) were irradiated with UV-B (500 J/m^2^—1 h) and analyzed as in (**b**). (**d**) Cells from (**b**) were incubated in EBSS medium (18 h) and analyzed as in (**b**). (**e**) Model of ZAKα activation by ribosome impairment, here caused by binding of anisomycin (ANI) to the peptidyl transferase center. The SAM domain inhibits activation whereas the kinase scans ribosomes (top panel—left). This inhibition is relieved upon recognition of ribotoxic stress signals by the C-terminal S and CTD domains (top panel—right). Kinase activation is signified by ZAKα autophosphorylation (P). Upon mutation of the SAM domain, sensing of ribotoxic stress by the RBR region (S + CTD) is not required for ZAKα activation (middle and bottom panels). ZAKα domains are color-coded as in (**a**). LZ, Leucine Zipper; SAM, Sterile Alpha Motif; YLD, YEATS-Like Domain; S, Sensor Domain; CTD, C-Terminal Domain; RBR, Ribosome Binding Region; and p-, phosphorylated (activated) form of kinase.

The outcome of the DALI analysis (Figure 3 and Figure 4 and Figure S1c and Table S1) prompted us to investigate the potential functional relevance of the YLD domain in ZAKα. We thus constructed a mutant version of ZAKα with a 50 amino acid deletion within the YLD (Δ434–484; ΔYLD) (Figure 5a). Through stable rescue in the U2OS ΔZAK background, we isolated two independent clones expressing this mutant at different levels. One clone (ΔYLD #1) expressed levels similar to endogenous ZAKα in WT U2OS cells, whereas the other clone (ΔYLD #2) expressed levels similar to exogenous ZAKα in our WT rescue cell line (Figure 5b). In both clones, ΔYLD supported the anisomycin-induced activation of p38 and JNK to a similar, but lower extent than WT ZAKα (Figure 5b). We also performed similar experiments with UV-B irradiation and EBSS starvation medium as sources of ribotoxic stress (Figure 5c,d). Moreover, here, the activation potential of ZAKα ΔYLD appeared to be diminished, but not absent, especially when assaying for p38 phosphorylation (Figure 5c,d). These results suggest that the YLD domain is dispensable for ZAKα activation after a range of ribotoxic insults but may be required for optimal functionality of the kinase.

## 4. Discussion

ZAKα is a ribotoxic stress-sensing kinase by virtue of its direct interaction with ribosomes. Unlike other ribosomal stress-surveying factors (e.g., GCN2 and ZNF598), ZAKα binds to elongating as well as stalled and collided ribosomes with apparent similar affinity [13,17,20]. These interactions are governed by the CTD and S domains located in the C-terminal part of ZAKα, with the CTD being more important for the “constitutive” or “scanning” mode of ribosome binding [20]. Upon ribotoxic stress, the two domains appear to be redundant for ZAKα activation, suggesting that impaired ribosomes present at least two different signals of structural aberrations that can be sensed by the kinase. Although the CTD appears to be an RNA-binding surface that displays affinity for helix 14 of 18S rRNA, located in the intersubunit ribosomal space, the structural determinants of the S domain and the nature of its interaction partner(s) have remained elusive. Here, we demonstrate that this domain contains an array of RGRYXXR/K peptide repeats that are seemingly redundant but require the positive charge of the arginines and lysines for functioning (Figure 1). 

These motifs are likely to form critical contacts with impaired ribosomes, but at present, we have no insight into whether this is based on interaction with rRNA or protein. Given the modular organization of the S domain (there are three redundant repeats) and the lack of an absolute requirement for this domain in the presence of the CTD, we speculate that impaired ribosomes expose a unique surface that is recognized by one RGRYXXR/K in a charge-dependent manner. Future work is required to identify this ribosomal binding surface, for example by cross-linking mass spectrometry or cryo-EM.

3D-homology modeling of a predicted folded domain in ZAKα revealed a hitherto ignored structural feature of this kinase. This domain, which we have dubbed the YLD, has a striking similarity to the well-characterized YEATS domain that is found in a number of nuclear proteins [33] (Figure 2, Figure 3 and Figure 4). YEATS domain containing proteins are involved in the establishment and reading of chromatin modifications, and YEATS domains are established protein-binding domains [33]. Notably, YEATS domains have been shown to bind modified and especially acetylated lysine residues [34,35,36,38]. It is thus likely that the YLD mediates interactions between ZAKα and other proteins or provides for intramolecular binding reactions with e.g., modified lysines. The regulation of such interactions is likely to impact on RSR signaling and may even underlie ZAKα kinase activation. Our partial 50 amino acid deletion mutant (Δ434–484) only mildly impaired RSR signaling, but the identification and mutation of critical residues in the domain will be required to firmly establish the importance of the YLD domain. One possibility is that the YLD assists ZAKα in its interactions with its relevant MAPKKs, such as MKK3 and MKK6 [20]. Interestingly, using mass spectrometry, acetylation has been reported on lysines both immediately upstream from and within the kinase domain of MKK6, but not in MKK3 (phosphosite.org (accessed on 16 March 2023)). Two such acetylation sites (K5 and K8) are found in the N-terminal D (Docking)-domain of MKK6 (phosphosite.org (accessed on 16 March 2023)), which is involved in the recognition of downstream p38 and JNK kinases [39]. MAPKKKs normally dock to the C-terminal Domain of Versatile Docking (DVD) of MAPKKs [40], but it is possible that ZAKα uses an alternative or additional mechanism to recognize MKK6 as its substrate. Further studies will be needed to elucidate the role that the ZAKα YLD plays in RSR signaling. 

Point mutation of the ZAKα SAM domain has been linked to mesoaxial polydactyly, hearing loss, and limb development in human patients as well as mouse models [19]. Our previous work surprisingly highlighted that these mutants display constitutive and ribotoxic stress-independent activity [20]. Thus, this developmental syndrome results from promiscuous RSR signaling rather than a defect in ZAKα activation. The described ZAKα SAM mutants also display decreased stability and thus, attenuated constitutive RSR signaling, potentially accounting for the non-lethal nature of the syndrome. In the present work, we highlight that the same mutants seemingly do not require communication with the ribosome for their activity (Figure 1f). These findings suggest that a functional SAM domain locks ZAKα in an inactive state that can only be circumvented by ribotoxic stress-sensing by the C-terminal S and CTD domains. We thus propose an allosteric ZAKα activation mechanism in which the SAM domain blocks the active site of the kinase domain (Figure 5e). When the sensor domains (S and CTD) are presented with ill-defined ribotoxic stress signals during ribosome scanning, conformational changes in the whole protein, and potentially the formation of other intramolecular interactions involving the SAM domain, unlock the kinase domain. In this model, the LZ and YLD do not play critical roles for ZAKα activation, which is consistent with our previous findings with LZ mutants [20] and the present findings with a partial YLD deletion mutant (Figure 5b–d).

## 5. Conclusions

In conclusion, our work highlights new structural features of the ZAKα kinase, improving our understanding of the mechanism(s) underlying RSR activation by impaired ribosomes. Structural information from ZAKα-bound ribosome complexes will be key to further inform our proposed model.

## Data Availability

JSON PAE files of the predicted ZAKα structure are available from the AlphaFold database: https://alphafold.ebi.ac.uk/ (accessed on 16 March 2023). Code is available at https://github.com/Valdemar-BI-Johansen/MAP3K20_PAE.git (accessed on 16 March 2023), and the array, which Figure 2b is based on, is also available in Table S2. The full results from our DALI analysis, including 1293 proteins, are available in an excel format as Supplementary Information in Table S1. The DALI method is accessible as a web service at http://ekhidna.biocenter.helsinki.fi/dali (accessed on 16 March 2023). The standalone version can be downloaded from http://ekhidna.biocenter.helsinki.fi/dali/#download (accessed on 16 March 2023).

## Supplementary Materials

The following supporting information can be downloaded at: https://www.mdpi.com/article/10.3390/cells12060969/s1, Figure S1: Distance-assisted matrix alignment (DALI) analysis; Table S1: DALI hits from comparison of amino acids 433–550 of ZAKα against the human AlphaFold database; and Table S2: JSON PAE amino acid array in excel format.

## References

[1] Hotamisligil G.S., Davis R.J. (2016). Cell Signaling and Stress Responses. Cold Spring Harb. Perspect. Biol..

[2] Shiba T., Ikeda M., Hara A., Yoshida H., Kaneko H., Takeuchi S. (1990). Mechanism of Acute Gastrointestinal Mucosal Damage in Endotoxic Shock and the Effect of Fragmin. Semin. Thromb. Hemost..

[3] Shiryaev A., Moens U. (2010). Mitogen-Activated Protein Kinase P38 and MK2, MK3 and MK5: Ménage à Trois or Ménage à Quatre?. Cell. Signal..

[4] Cuadrado A., Nebreda A.R. (2010). Mechanisms and Functions of P38 MAPK Signalling. Biochem. J..

[5] Joshi S., Platanias L.C. (2014). Mnk Kinase Pathway: Cellular Functions and Biological Outcomes. World J. Biol. Chem..

[6] Manke I.A., Nguyen A., Lim D., Stewart M.Q., Elia A.E.H., Yaffe M.B. (2005). MAPKAP Kinase-2 Is a Cell Cycle Checkpoint Kinase That Regulates the G2/M Transition and S Phase Progression in Response to UV Irradiation. Mol. Cell.

[7] Canovas B., Nebreda A.R. (2021). Diversity and Versatility of P38 Kinase Signalling in Health and Disease. Nat. Rev. Mol. Cell Biol..

[8] Borisova M.E., Voigt A., Tollenaere M.A.X., Sahu S.K., Juretschke T., Kreim N., Mailand N., Choudhary C., Bekker-Jensen S., Akutsu M. (2018). P38-MK2 Signaling Axis Regulates RNA Metabolism after UV-Light-Induced DNA Damage. Nat. Commun..

[9] Tollenaere M.A.X., Villumsen B.H., Blasius M., Nielsen J.C., Wagner S.A., Bartek J., Beli P., Mailand N., Bekker-Jensen S. (2015). P38- and MK2-Dependent Signalling Promotes Stress-Induced Centriolar Satellite Remodelling via 14-3-3-Dependent Sequestration of CEP131/AZI1. Nat. Commun..

[10] Dhanasekaran D.N., Reddy E.P. (2017). JNK-Signaling: A Multiplexing Hub in Programmed Cell Death. Genes Cancer.

[11] Semba T., Sammons R., Wang X., Xie X., Dalby K.N., Ueno N.T. (2020). JNK Signaling in Stem Cell Self-Renewal and Differentiation. Int. J. Mol. Sci..

[12] Nikolic I., Leiva M., Sabio G. (2020). The Role of Stress Kinases in Metabolic Disease. Nat. Rev. Endocrinol..

[13] Vind A.C., Genzor A.V., Bekker-Jensen S. (2020). Ribosomal Stress-Surveillance: Three Pathways Is a Magic Number. Nucleic Acids Res..

[14] Grollman A.P. (1967). Inhibitors of Protein Biosynthesis: II. Mode of Action of Anisomycin. J. Biol. Chem..

[15] Walsh M.J., Dodd J.E., Hautbergue G.M. (2013). Ribosome-Inactivating Proteins: Potent Poisons and Molecular Tools. Virulence.

[16] Iordanov M.S., Pribnow D., Magun J.L., Dinh T.H., Pearson J.A., Magun B.E. (1998). Ultraviolet Radiation Triggers the Ribotoxic Stress Response in Mammalian Cells. J. Biol. Chem..

[17] Wu C.C.-C., Peterson A., Zinshteyn B., Regot S., Green R. (2020). Ribosome Collisions Trigger General Stress Responses to Regulate Cell Fate. Cell.

[18] Snieckute G., Genzor A.V., Vind A.C., Ryder L., Stoneley M., Chamois S., Dreos R., Nordgaard C., Sass F., Blasius M. (2022). Ribosome Stalling Is a Signal for Metabolic Regulation by the Ribotoxic Stress Response. Cell Metab..

[19] Spielmann M., Kakar N., Tayebi N., Leettola C., Nürnberg G., Sowada N., Lupiáñez D.G., Harabula I., Flöttmann R., Horn D. (2016). Exome Sequencing and CRISPR/Cas Genome Editing Identify Mutations of ZAK as a Cause of Limb Defects in Humans and Mice. Genome Res..

[20] Vind A.C., Snieckute G., Blasius M., Tiedje C., Krogh N., Bekker-Jensen D.B., Andersen K.L., Nordgaard C., Tollenaere M.A.X., Lund A.H. (2020). ZAKα Recognizes Stalled Ribosomes through Partially Redundant Sensor Domains. Mol. Cell.

[21] Holm L., Rosenström P. (2010). Dali Server: Conservation Mapping in 3D. Nucleic Acids Res..

[22] Holm L., Laakso L.M. (2016). Dali Server Update. Nucleic Acids Res..

[23] Holm L., Laiho A., Törönen P., Salgado M. (2023). DALI Shines a Light on Remote Homologs: One Hundred Discoveries. Protein Sci..

[24] Holm L., Gáspári Z. (2020). Using Dali for Protein Structure Comparison. Structural Bioinformatics: Methods and Protocols.

[25] Nakai N., Kitai S., Iida N., Inoue S., Higashida K. (2020). Autophagy under Glucose Starvation Enhances Protein Translation Initiation in Response to Re-addition of Glucose in C2C12 Myotubes. FEBS Open Bio.

[26] Larsen S.C., Sylvestersen K.B., Mund A., Lyon D., Mullari M., Madsen M.V., Daniel J.A., Jensen L.J., Nielsen M.L. (2016). Proteome-Wide Analysis of Arginine Monomethylation Reveals Widespread Occurrence in Human Cells. Sci. Signal..

[27] Mathea S., Abdul Azeez K.R., Salah E., Tallant C., Wolfreys F., Konietzny R., Fischer R., Lou H.J., Brennan P.E., Schnapp G. (2016). Structure of the Human Protein Kinase ZAK in Complex with Vemurafenib. ACS Chem. Biol..

[28] Chang Y., Lu X., Shibu M.A., Dai Y.-B., Luo J., Zhang Y., Li Y., Zhao P., Zhang Z., Xu Y. (2017). Structure Based Design of N-(3-((1H-Pyrazolo[3,4-b]Pyridin-5-Yl)Ethynyl)Benzenesulfonamides as Selective Leucine-Zipper and Sterile-α Motif Kinase (ZAK) Inhibitors. J. Med. Chem..

[29] Yang J., Shibu M.A., Kong L., Luo J., BadrealamKhan F., Huang Y., Tu Z.-C., Yun C.-H., Huang C.-Y., Ding K. (2020). Design, Synthesis, and Structure–Activity Relationships of 1,2,3-Triazole Benzenesulfonamides as New Selective Leucine-Zipper and Sterile-α Motif Kinase (ZAK) Inhibitors. J. Med. Chem..

[30] Jumper J., Evans R., Pritzel A., Green T., Figurnov M., Ronneberger O., Tunyasuvunakool K., Bates R., Žídek A., Potapenko A. (2021). Highly Accurate Protein Structure Prediction with AlphaFold. Nature.

[31] Varadi M., Anyango S., Deshpande M., Nair S., Natassia C., Yordanova G., Yuan D., Stroe O., Wood G., Laydon A. (2022). AlphaFold Protein Structure Database: Massively Expanding the Structural Coverage of Protein-Sequence Space with High-Accuracy Models. Nucleic Acids Res..

[32] Wang A.Y., Schulze J.M., Skordalakes E., Gin J.W., Berger J.M., Rine J., Kobor M.S. (2009). Asf1-like Structure of the Conserved Yaf9 YEATS Domain and Role in H2A.Z Deposition and Acetylation. Proc. Natl. Acad. Sci. USA.

[33] Schulze J.M., Wang A.Y., Kobor M.S. (2009). YEATS Domain Proteins: A Diverse Family with Many Links to Chromatin Modification and Transcription. Biochem. Cell Biol..

[34] Li Y., Wen H., Xi Y., Tanaka K., Wang H., Peng D., Ren Y., Jin Q., Dent S.Y.R., Li W. (2014). AF9 YEATS Domain Links Histone Acetylation to DOT1L-Mediated H3K79 Methylation. Cell.

[35] Li Y., Sabari B.R., Panchenko T., Wen H., Zhao D., Guan H., Wan L., Huang H., Tang Z., Zhao Y. (2016). Molecular Coupling of Histone Crotonylation and Active Transcription by AF9 YEATS Domain. Mol. Cell.

[36] Andrews F.H., Shinsky S.A., Shanle E.K., Bridgers J.B., Gest A., Tsun I.K., Krajewski K., Shi X., Strahl B.D., Kutateladze T.G. (2016). The Taf14 YEATS Domain Is a Reader of Histone Crotonylation. Nat. Chem. Biol..

[37] Wang W., Xue Y., Zhou S., Kuo A., Cairns B.R., Crabtree G.R. (1996). Diversity and Specialization of Mammalian SWI/SNF Complexes. Genes Dev..

[38] Zhao D., Li Y., Xiong X., Chen Z., Li H. (2017). YEATS Domain-A Histone Acylation Reader in Health and Disease. J. Mol. Biol..

[39] Cargnello M., Roux P.P. (2011). Activation and Function of the MAPKs and Their Substrates, the MAPK-Activated Protein Kinases. Microbiol. Mol. Biol. Rev..

[40] Gaestel M., Kracht M. (2009). Peptides as Signaling Inhibitors for Mammalian MAP Kinase Cascades. Curr. Pharm. Des..

