# Supplementary material for: Computational and Functional Analysis of Structural Features in the ZAKα Kinase
Source: Cells. 2023 Mar 22;12(6):969. doi: 10.3390/cells12060969 (PMC10047201; doi:10.3390/cells12060969)
Supplement: Supplementary file 1 [file cells-12-00969-s001.zip › Supplementary Material.pdf]

cells

Supplementary Materials for

Computational and functional analysis of structural features in the ZAKα kinase

Valdemar Brimnes Ingemann Johansen [1], Goda Snieckute [1], Anna Constance Vind [1], Melanie Blasius [1], Simon Bekker-Jensen [1,*]

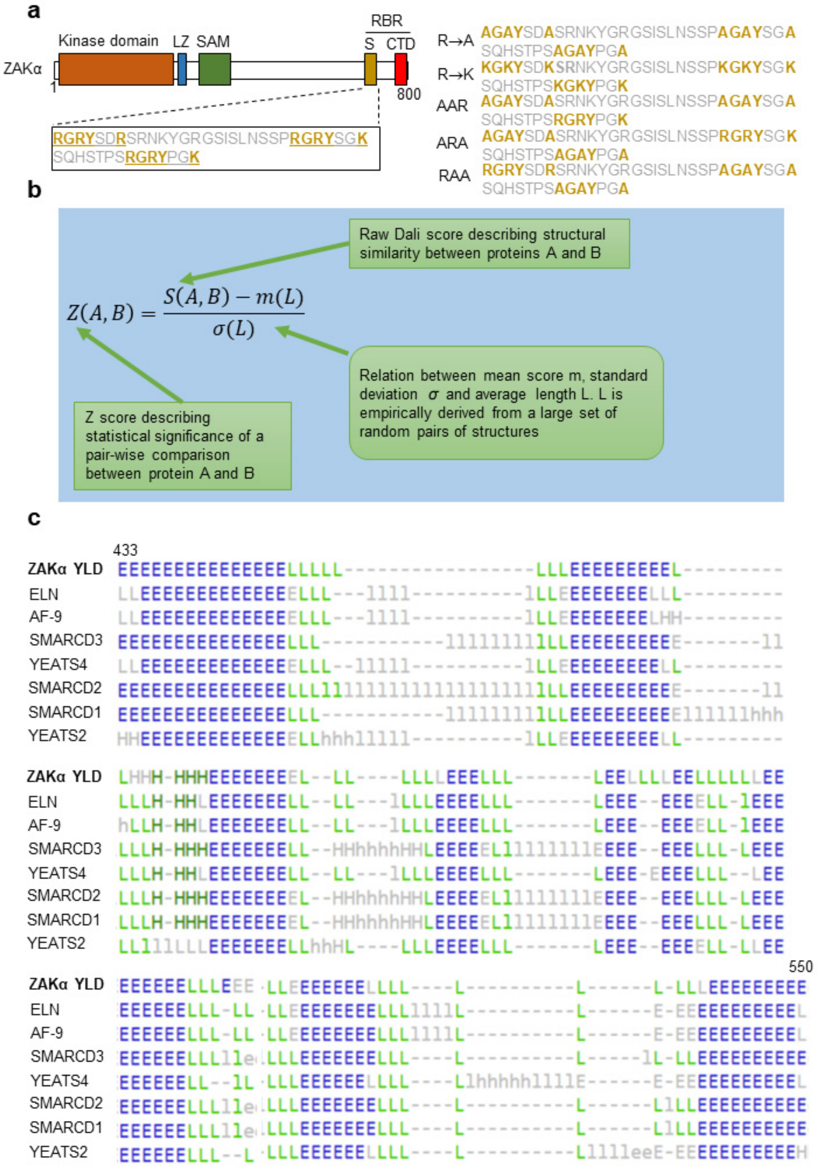

Figure S1. Distance-assisted matrix alignment (DALI) analysis. (**a**) Schematic of the ZAKα protein highlighting the three RGRYXXR/K motifs in the sensor (S) domain. Amino acid sequences of constructed mutants in this region (R -> A, R -> K, AAR, ARA and RAA) are indicated. LZ, Leucine Zipper; SAM, Sterile Alpha Motif; S, Sensor Domain; CTD, C-Terminal Domain; RBR, Ribosome

Binding Region. (**b**) To assign statistical significance to our pair-wise comparison score (S(A,B)), we employed the Z-score (Z(A,B)) developed by Holm and colleagues (27). (**c**) Secondary sequence alignment of ZAKα YLD and the DALI hits from Figure 3b-i. Gaps indicate unaligned regions. Uppercase letters denote structurally equivalent positions with ZAKα YLD, whereas lowercase letters denote insertions relative to ZAKα YLD. The most frequent secondary structure to which a specific amino acid residue contributes at a specific position is colored in each column. H/h, helix, E/e, strand, L/l, coil.

Table S1. DALI hits from comparison of amino acids 433-550 of ZAK*α* against the human AlphaFold database. DALI hits with Z scores above or equal 2 are shown.

Table S2. JSON PAE amino acid array in excel format. The JSON PAE file can be downloaded from https://alphafold.ebi.ac.uk/, and the code can be accessed via https://github.com/Valdemar-BI-Johansen/MAP3K20_PAE.git.
